# Incretin-based therapies and PPARγ agonists as regulators of adipokines-Nrf2 axis in diabetic cardiovascular disease

**DOI:** 10.21542/gcsp.2026.20

**Published:** 2026-06-30

**Authors:** Israa O. Kashmoola, Shatha H. Mohammad, Mohammad H. Alsaaty

**Affiliations:** 1 Nineveh Health Directorate, Mosul, Nineveh Province, 41006, Iraq; 2 University of Mosul, College of Medicine, Department of Pharmacology, Mosul, Nineveh Province, 41002, Iraq; 3 University of Mosul, College of Medicine, Department of Medicine, Mosul, Nineveh Province, 41002, Iraq

## Abstract

Oxidative stress and adipokine imbalance are important contributors to the pathogenesis of diabetic cardiovascular disease. The nuclear factor erythroid 2-related factor 2 (Nrf2) regulates antioxidant defense, while adipokines link metabolism and inflammation. Incretin-based therapies and PPARγ agonists may join on these pathways to provide cardiovascular protection beyond glycemic control. This review aims to investigate the current evidence on how incretin-based agents and PPARγ agonists regulate the adipokine-Nrf2 axis and their impact on cardiovascular outcomes in diabetes. A literature search was performed using PubMed, Google Scholar and Scopus to include reviews, experimental, clinical, and translational studies published in English until November 2025. Evidence indicates that incretin-based agents and PPARγ agonists synergistically activate Nrf2 and inhibit NF-kB signaling, leading to improved oxidative status and favorable adipokine levels. Increased adiponectin and omentin, and suppressed resistin, leptin and TNF-α contribute to reduced inflammation and enhanced vascular and myocardial protection. Collectively, combined activation of incretin and PPARγ pathways modulates the adipokine-Nrf2 axis, offering joined antioxidant and anti-inflammatory benefits that may reduce diabetic cardiovascular risk.

## Introduction

Oxidative stress is one of the main determinants of type 2 diabetes mellitus (T2DM) complications. It has a key pathophysiological responsibility in the initiation and progression of heart and vascular illnesses^1^. Activation of anti-oxidant defense is essential to prevent or postpone the development of diabetic-cardiovascular complications^2^.

Nuclear factor (erythroid-derived 2)-like 2 (Nrf2), a master transcription factor which is activated by increased reactive oxygen species (ROS), is found in most tissues and exerts a major role in augmentation of the antioxidant pathways, resulting in increased endothelial protection^3^.

Beside oxidative stress, alterations in adipokines secretion, characterized by reduced protective mediators as adiponectin and elevated pro-inflammatory factors, can further predispose to increased susceptibility of cardiovascular disease (CVD)^4^. Accordingly, the choice of appropriate medications in T2DM that modulate adipokine and oxidative stress is critically important and considered as one of the major determinants of patient’s cardiac health.

Incretin-based drugs, such as GLP-1 agonists and DPP4 inhibitors, are widely used alongside metformin for diabetic patients. Added to their hypoglycemic effect, incretin agonists may provide antioxidative, anti-inflammatory, and vascular protecting potential in diverse tissues including heart and blood vessels^5^. Similarly, thiazolidinediones (TZD), which work by activation of peroxisome proliferator-activated receptor gamma (PPARγ), may give a vascular protection due to their ability to increase lipid and glucose uptake, and lower free fatty acid levels, and insulin resistance^6^.

Oxidative stress and inflammation are recognized factors in diabetic CVD; however, the comprehensive function of adipokines within Nrf2-centric regulatory networks is not fully elucidated. Moreover, the mechanisms by which incretin and PPARγ signaling converge on this axis to affect cardiovascular outcomes have not been systematically postulated.

To address this gap, we suggest an integrative framework that illustrates the adipokine-Nrf2 axis as a two-way regulatory network to connect metabolic signals, oxidative stress responses, and inflammatory pathways. In this context, protective adipokines, such as adiponectin, may augment Nrf2 transcriptional activity while inhibiting NF-kB-mediated inflammation.

**Figure 1.**
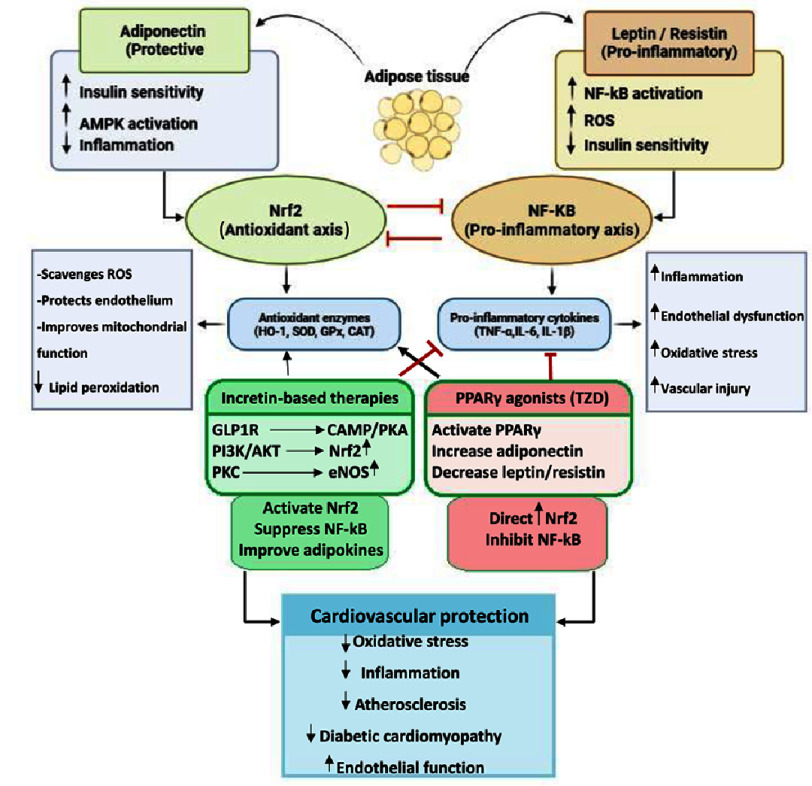
Integrative model of the adipokine-Nrf2-NF-kB axis and its modulation by incretin signaling and PPARγ activation in diabetic cardiovascular disease. An integrative schematic demonstrating the bidirectional interactions among adipokines, oxidative stress, and inflammatory signaling pathways in diabetic cardiovascular disease. Protective adipokines, especially adiponectin, stimulate AMPK and promote Nrf2 signaling. This causes antioxidant defense systems function better and NF-kB-mediated inflammation to be decreased. On the other hand, pro-inflammatory adipokines like leptin and resistin increase the production of ROS and turn on NF-kB signaling, which makes inflammatory responses stronger and stops Nrf2 from working properly. Incretin-based therapies (GLP-1 receptor agonists and DPP-4 inhibitors) and PPARγ agonists influence this network *via* upstream pathways, such as PI3K/AKT, cAMP, and AMPK signaling. These interventions boost Nrf2 transcriptional activity, lower oxidative stress, and lessen inflammatory signaling. Therefore, they help the endothelium work better and protect the heart and blood vessels. Solid arrows indicate activation or stimulation, upward and downward arrows mean relative increase and decrease, respectively, whereas blunt-ended lines represent inhibitory or suppressive effects. Nrf2, Nuclear factor erythroid 2-related factor 2; NF-kB, Nuclear factor kappa B; IL-6, Interleukin-6; TNF-α, Tumor necrosis factor alpha; IL-1β, Interleukin-1 beta; HO-1, Heme oxygenase-1; SOD, Superoxide dismutase; GPx, Glutathione peroxidase; CAT, Catalase; AMPK, AMP-activated protein kinase; PPARγ, Peroxisome proliferator-activated receptor gamma; GLP-1, Glucagon-like peptide-1; DPP-4, Dipeptidyl peptidase-4; PI3K, Phosphoinositide 3-kinase; AKT, Protein kinase B; cAMP, Cyclic adenosine monophosphate; ROS, Reactive oxygen species; eNOS, Endothelial nitric oxide synthase.

On the other hand, pro-inflammatory adipokines like leptin and resistin are linked to the activation of NF-kB, the production of more ROS, and the loss of function of Nrf2 signaling. Moreover, incretin-based therapies and PPARγ agonists seem to alter this network *via* upstream pathways like PI3K/AKT and AMPK, affecting redox balance and inflammatory tone, which leads to cardiovascular protection. Although these interactions have been demonstrated by experimental and translational studies, they have not yet been comprehensively outlined within a cohesive mechanistic framework. Figure 1 shows a visual representation of this proposed axis.

This review examines contemporary evidence regarding the convergence of incretin-based therapies and PPARγ signaling on Nrf2 pathways and adipokine regulation to influence oxidative stress in diabetic CVD, focusing on their potential translational significance as therapeutic targets.

### Cardiovascular status in diabetic patients

Cardiovascular health in T2DM is critically compromised due to increased risk of various cardiovascular events, such as cardiomyopathy, atherosclerosis, heart attacks, strokes, heart failure and cardiac metabolic abnormalities^7^.

Diabetic cardiomyopathy is associated with structural and functional abnormalities ranging from cardiac fibrosis, inflammation, increased apoptosis, and myocardial lipid accumulation, to left ventricular mass formation, reduced contractile ability, and impaired systolic and diastolic functions^8^.

The presence of various risk factors in diabetic patients, such as obesity, hypertension, and dyslipidemia may further exacerbate cardiac complications. The accelerated cardiovascular damage induced by hyperglycemia may be compounded by increased oxidative stress, inflammation and dysregulation of adipokines.

Through several mechanisms—including protein and lipid peroxidation, DNA damage, oxidative modification of microRNAs, and activation of stress-sensitive pathways—excessive reactive oxygen species (ROS) cause cellular dysfunction and injury^9^.

Through elevated production of TNF-α, NF-κB, and collagen factors, prolonged exposure to oxidative stress in T2DM patients can cause chronic inflammation and fibrosis in the heart and blood vessels^10^.

Understanding these pathophysiological pathways is important for identifying molecular targets such as PPARγ signaling, incretin pathways and Nrf2 regulation to restore metabolic and antioxidant homeostasis for improved cardiac outcome in diabetes.

### Adipokines in metabolic and cardiovascular regulation

Adipocytokines are secretory bioactive substances produced by adipose tissue to directly affect insulin resistance (IR), angiogenesis, inflammation, in addition to glucose and lipid metabolism. Fibroblasts, macrophages, lymphocytes, neutrophils, mast cells, endothelial cells, and adipocyte precursors have also been reported to produce adipokines^11^.

One of the major adipokines is adiponectin, which has antiatherogenic, anti-inflammatory, antidiabetic, immunomodulatory, antiapoptotic and vasoprotective properties^12^. Low adiponectin levels are connected to a higher incidence of obesity-related CVD, such as peripheral artery disease and ischemic heart disease^13^. According to experimental results, adiponectin directly affects the constituent cells of the heart and blood vessels to have positive effects on the cardiovascular system (CVS)^14^. Adiponectin guards cardiovascular cells under situations of stress through stimulation of endothelial cell responses and inhibition of hypertrophic and pro-inflammatory responses^15^.

Apelin, as a protective adipokine, has beneficial abilities in the management of IR, sclerosis, gastrointestinal tract inflammation, hypertension, neuropathic pain, beside hepatic acute kidney, and lung injuries^16–18^. The potential application of apelin as a therapeutic treatment in heart failure is made possible by its vasodilator and inotropic properties, as well as its upregulation following positive left ventricular remodeling. Nevertheless, there is evidence that apelin can occasionally behave as a pro-inflammatory factor, inhibiting activated macrophage capabilities and promoting the release of inflammatory cytokines^19^.

Similarly, omentin (intelectin) possesses beneficial effects such as anti-inflammatory, anti-atherogenic, insulin-sensitizing, cardio-protective, and oxidative stress-decreasing effects. enhanced endothelial cell function and survival, augmented endothelial nitric oxide synthase and nitric oxide (NO) bioavailability, improved vascular smooth muscle cell relaxation with decreased proliferation, and suppressed inflammation, and oxidation are the reasons behind omentin’s cardiovascular protective effects. This makes omentin an excellent therapeutic option in diabetes mellitus and cardiovascular and inflammatory diseases^20,21^.

Another adipokine that influences the specification of cardiovascular tissues both during and after development is retinoid acid receptor responder protein 2 (RARRP2). According to recent research, RARRP2 plays a significant part in the process of cardiac remodeling in rats with hypertension and after myocardial infarction^22^. Many biological activities of RARRP2 are provoked by the ligand-activated transcription factors and it can activate PPARβ/*δ* to enhance lipolysis. RARRP2 treatment has shown to restore adipose PPARβ/*δ* expression; therefore, may provide a valuable choice in the controlling and prevention of the metabolic syndrome^23^.

On the other hand, leptin shows a pro-inflammatory action, causing early development of hyperphagia, hypogonadism, obesity, and metabolic disorders^24^. Leptin-mediated boosts of blood pressure and heart rate may aggravate cardiac hypertrophy and myocardial workload in the long term^25^.

Resistin, another adipokine, is importantly expressed in macrophages and has been shown to be implicated in inflammation, diabetes and obesity^26^. Regarding CVD and atherosclerosis, resistin has been reported to participate in the progress of angiogenesis thrombosis, and endothelial and smooth muscle cell dysfunction^27^. Similar to resistin, visfatin has shown increased levels in obesity, where high levels of it appear to be linked with increased inflammation and may contribute to the development of T2DM, IR, CVD and renal complications^28,29^.

Numerous disorders have been linked to chemerin, a pleiotropic factor implicated in inflammation, adipogenesis, angiogenesis, and energy metabolism^30^. An elevated risk of coronary artery disease was linked to higher serum chemerin levels. Chemerin may therefore constitute a unique connection between metabolic signals and atherosclerosis^31^.

Likewise, the liver and adipose tissue produce the adipokine retinol binding protein-4 (RBP4). Apart from its function in retinol transport, new research indicates that RBP4 not only directly damages cardiomyocytes, but also plays a role in the pathophysiology of heart failure by causing IR and chronic inflammation^32^. Dipeptidyl peptidase-4 (DPP4) has emerged as an adipokine that is secreted by adipose tissue and work as a multifactorial enzyme to regulate glucose, lipid, insulin and inflammation^33^. Studies show that elevated levels of DPP4 is associated with obesity, IR, T2DM and CVD^34,35^.

TNF-α is highly expressed in the failing heart, owing not only to chemical mediators released as a result of arterial occlusion, but also to its direct depressant effect on myocyte contractility. The elevation of TNF-α may promote endothelial dysfunction, oxidative stress, and apoptosis of cardiomyocytes^36^.

Likewise, interleukin-6 (IL-6), a pivotal cytokine of innate immunity, is considered as a proatherogenic factor in CVD^37,38^.

One of the adipokines that has direct effect on cardiomyocyte size and number is lipocalin-2 (LCN2). It is believed to play a central role in cardiac hypertrophy, heart failure and T2DM^39,40^. Table 1 summarizes the proposed mechanism by which different adipokines provide cardiovascular benefits.

**Table 1:** Summary of the effect of main adipokines on Nrf2/NF-kB axis and cardiovascular outcomes.

**Adipokine**	**Effect on Nrf2/NF-kB**	**Cardiovascular impact**
**Adiponectin**	Activates AMPK–Nrf2, suppresses NF-kB	Anti-atherogenic, anti-inflammatory, improves endothelial function
**Apelin**	Activates protective pathways, can sometimes act pro-inflammatory	Vasodilatory, inotropic, potential therapeutic in HF
**Omentin**	Activates Nrf2, suppresses NF-kB, increases NO bioavailability	Cardioprotective, improves endothelial survival and function
**Leptin**	Activates NF-kB, increases ROS, suppresses Nrf2	Pro-hypertrophic, raises BP and HR, worsens CV outcomes
**Resistin**	Activates NF-kB, promotes inflammation, suppresses Nrf2	Promotes endothelial dysfunction, thrombosis, smooth muscle dysfunction
**Visfatin**	Activates NF-kB when elevated, promotes inflammation	Associated with IR, diabetes, CVD, promotes vascular inflammation
**DPP4**	Promotes NF-kB–mediated inflammation; indirectly suppresses Nrf2 activity through oxidative stress pathways	Linked to endothelial dysfunction and increased cardiovascular risk; inhibition improves vascular function and reduces inflammation
**TNF-α**	Strong NF-kB activator, suppresses Nrf2	Induces oxidative stress, apoptosis, poor cardiac outcomes
**IL-6**	Pro-inflammatory, NF-kB activator	Contributes to atherogenesis, vascular dysfunction
**RBP4**	Promotes inflammation, insulin resistance, cardiomyocyte injury	Linked to HF progression, insulin resistance, inflammation
**Chemerin**	Linked with atherosclerosis, promotes inflammation	Associated with CAD risk, metabolic dysregulation
**LCN2**	Associated with cardiac hypertrophy, pro-inflammatory	Elevated in T2DM, linked to hypertrophy and HF

### Effect of dysregulated adipokines on cardiovascular health and diabetes

Under normal circumstances, the production of proinflammatory adipokines (leptin, visfatin, resistin and RBP4) is in balance with anti-inflammatory adipokines (omentin, apelin, adiponectin)^41^. Obesity disrupts the normal balance between the two classes; however, it is worth knowing the difference between the metabolic function of central (visceral abdominal) and peripheral obesity (subcutaneous) in the production of adipokines^42^.

Visceral obesity is linked with high levels of some proinflammatory adipokines, leading to metabolic disorders. Overloaded adipocytes with proinflammatory adipokines produce excess energy-rich molecules and trigger cellular stress, causing chronic low-grade inflammation and serious negative cardiovascular consequences such as arterial stiffness, altered lipid metabolism, and IR^43^.

### Nrf2 signaling and vascular protection in diabetes

By triggering the transcription of several antioxidant genes, Nrf2 controls the cellular antioxidant defense system and fights oxidative stress brought on by diabetes^44^. Recent reports suggest that activation of Nrf2 may provide protection against cardiovascular metabolic disorders^45^. The aryl hydrocarbon receptor (AhR), PPARγ or PPARα, specificity protein 1 (Sp-1), p53, NF-κB, myocyte-specific enhancer factor 2 D (MEF2D), breast cancer 1 (BRCA1), c-Jun, and c-Myc, are among the transcription factors that typically activate Nrf2 transcription. Nrf2 transcriptional control is also influenced by epigenetic processes, such as methylation of the Nrf2 promoter in CpG islands or acetylation of H4 histone and H3 histone^46,47^.

A number of miRNAs can downregulate Nrf2 production at the post-transcriptional stage. However, Kelch-like ECH-associated protein 1 (KEAP1) primarily controls the transcriptional activity and protein stability of Nrf2^48^. Nrf2 can also be activated by structural inhibition of KEAP1. On the one hand, KEAP1 prevents Nrf2 from translocating to the nucleus; on the other, it promotes Nrf2’s proteasomal breakdown.

Since the protective effect has been confirmed in animal models of metabolic CVD, small molecule-induced structural inhibition of KEAP1 protein - a canonical method of activating Nrf2 - has become a hot research topic in recent decades^49^.

Other methods have been used to activate Nrf2 in metabolic CVD in addition to structural suppression of the KEAP1 protein. These include Nfe2l2 gene transcription activation, KEAP1 protein level reduction through microRNA-induced KEAP1 mRNA degradation, inhibition of Nrf2 protein proteasomal degradation, and modification of additional upstream Nrf2 regulators^50^.

When cardiac autophagy is normal, Nrf2 is essential for cardiac adaptability; when myocardial autophagy is compromised in pressure-overloaded hearts, it exaggerates cardiac pathological decompensation. Therefore, patients who have low Nrf2 levels in various tissues are probably at risk to a number of detrimental aspects of the development of the disease. Nrf2 expression is probably insufficient to prevent oxidative stress, atherosclerosis, and heart failure if it is not enough to prevent hypertension^51^.

### Consequence of the antagonistic relationship between Nrf2, adipokines and inflammation

Clinical trials have identified oxidative stress and chronic inflammatory conditions as major drivers for cardiovascular risks. Atherosclerotic disorders and CVD are directly linked with inflammation due its interaction with oxidative stress^52^. Nrf2 regulate antioxidant and cytoprotective properties, while NF-kB act as a master controller of pro-inflammatory responses. Available evidence documents an inverse relationship between inflammation and Nrf2, validating the antagonistic behavior in the heart^53^. Additionally, adipokines have emerged as upstream modulators of this axis, linking metabolic status to inflammation and oxidative stress.

Under normal physiology, Nrf2 protects cardiomyocytes and vascular endothelial cells *via* activation of antioxidant response elements (AREs), controlling the representation of antioxidant enzymes that antagonize ROS accumulation and suppress cardiac oxidative injury^54^. Conversely, NF-kB stimulation by cytokines, AGEs, and oxidative stress induces transcription of pro-inflammatory adipokines including TNF-α and IL-6, causing inflammation and oxidative injury to the heart and blood vessels^55^.

Failure of Nrf2 defense predispose to endothelial dysfunction, foam cell formation, plaque instability, hypertrophy, fibrosis, contractile dysfunction and heart failure^56^. Also, during ischemia, in adequate activation of Nrf2 may exacerbate ROS-mediated apoptosis and necrosis of cardiac cells^57^. Adipokines participate in this axis through their influence on systemic inflammation, metabolism, and cardiovascular health by regulating both Nrf2 and NF-kB activity^51^.

Protective effects are driven by adiponectin, omentin, and apelin *via* activation of Nrf2 signaling, promoting antioxidant expression and suppressing inflammation. Reports have shown that adiponectin may activate AMPK-Nrf2 pathway, improving endothelial NO generation and protecting against myocardial ischemic injury.

Conversely, excessive amounts of pro-inflammatory adipokines activate NF-kB signaling, promoting ROS production and suppression of Nrf2 activity, which triggers vascular inflammation, myocardial injury and may progress to heart^58–60^.

### Incretin-based therapy and cardiovascular protection

The known incretin hormones from the upper (GIP, K cells) and lower (GLP-1, L cells) guts are GIP and GLP-1. In order to lower blood glucose levels, GLP-1 binds to a G-protein coupled receptor in the pancreas, enhances insulin secretion from pancreatic β-cells, decreases glucagon release from α-cells, and stimulates the creation of adenylyl cyclase and cyclic adenosine monophosphate (cAMP)^61^. Furthermore, it decreases the motility and emptying of the stomach, in addition to increasing the sensing of satiety through its hypothalamic action^62^.

DPP-4 quickly breaks down circulating GLP-1 to GLP-1 amide. GLP-1 receptor agonists (GLP-1RAs) and dipeptidyl peptidase-4 inhibitors (DPP-4is) are the two primary families of incretin antidiabetic medications that have been produced. In addition to their antidiabetic properties, incretin-based medications benefit a number of bodily functions^63^. The breakdown of other peptides that are substrates to DPP-4, such as the gastric inhibitory polypeptide (GIP) and a range of chemokines are also decreased by DPP-4is^64^.

Research has demonstrated that incretin base therapies offer cardioprotective benefits through direct effects on the heart and vasculature as well as glycemic and non-glycemic effects, such as improved insulin secretion and action, body weight loss, blood pressure lowering, and improved lipid profile. These effects, especially in those with T2DM and proved atherosclerotic cardiovascular disease, are probably coupled with anti-inflammatory and antioxidant qualities that result in decreases in atherothrombotic events^65,66^.

GLP-1RAs, in contrast to DPP-4is, may affect obesity and chronic renal disease, illnesses for which there are few alternatives for lowering cardiovascular risk. A modulatory impact at the carotid sinus level has been seen in animal models, indicating that this pharmacological class may affect the control of sympathetic tone under hyperglycemic conditions^67^.

Numerous cardiovascular risk factors, such as obesity, dyslipidemia, and hypertension, raise the risk of atherosclerosis and associated consequences in T2DM patients. It has been demonstrated that GLP-1-based treatments lower systolic blood pressure by 2 to six mm Hg, which mediates a decrease in cardiovascular events^68^.

GLP-1 and GLP-1 RAs reduce the development and progression of atherosclerotic lesions by producing more stable, less susceptible plaques, according to a variety of experimental data in preclinical models of atherosclerosis. This is most likely due to their antiatherogenic and anti-inflammatory effects in endothelial cells, monocytes, macrophages, and vascular smooth muscle cells^69^.

In addition to their metabolic effects, recent clinical and experimental findings indicate that DPP-4is and GLP-1RAs modulate various stages of the cardiovascular cycle, including cardiovascular risk factors, molecular mechanisms involved in atherogenesis, ischemic heart disease, and heart failure. Thus, the use of GLP-1RAs and DPP-4is may be a new strategy to affect cardiovascular disease in T2DM patients^64^.

Furthermore, GLP-1RAs and DPP-4is exhibit antioxidative effects through receptor-mediated activation of the PKC, cAMP, and PI3K pathways as well as Nrf-2. It has been demonstrated that GLP-1 activation reduced oxidative damage in mitochondria by restoring cytochrome c oxidase activity and mitochondrial membrane permeability. In neonatal rat cardiomyocytes, exendin-4 treatment reduced ROS generation, induced by hydrogen peroxide, and boosted the synthesis of antioxidant enzymes like catalase, manganese superoxide dismutase and glutathione peroxidase-1. This effect was dependent on GLP-1R-mediated Epac pathways^70,71^.

### PPAR and cardiovascular protection

It has been demonstrated that PPARs are crucial in metabolic disorders such obesity, insulin resistance, and coronary artery disease. There are three known subtypes of PPAR receptors: PPARα, PPAR*δ*/β, and PPARγ.

The liver, muscle, kidney, and heart all contain PPARα. Although PPAR*δ*/β is expressed in numerous tissues, it is most prominent in the brain, skin, and adipose tissues^72^. PPARγ presents in a high level in fat, with low level presented in the liver, and very low amount in the muscle.

The synthetic activators of PPARγ, thiazolidinediones (TZDs), have been shown to exert cardiovascular protection through several mechanisms. They improve insulin sensitivity, reduce circulating free fatty acids, and attenuate oxidative stress and inflammation within the cardiovascular system^73^. Additionally, TZDs can suppress pro-inflammatory cytokine expression and inhibit vascular smooth muscle cells proliferation. Furthermore, activation of PPARγ by TZDs results in enhancement of endothelial NO bioavailability; thereby improving vascular function, and maintaining cardiovascular health. Moreover, when PPAR-γ is activated, anti-hyperglycemic adipokines are secreted and non-esterified fatty acids are deposited in adipose tissue rather than the liver and skeletal muscle^74,75^.

Adiponectin has the ability to raise PPAR-γ in adipose tissue, which improves the tissue’s insulin sensitivity and anti-inflammatory properties^76^. The Nrf2 and PPARγ pathways appear to be linked by a positive feedback loop that simultaneously sustains the production of transcription factors and their target antioxidant genes^77^. Huang et al. have identified PPARγ as a valuable target gene provoked by transcriptional activation of Nrf2^78^. Several other researchers have also observed direct binding of Nrf2 to recently discovered AREs in the PPARγ promoter regions.

Finding the optimal combination of Nrf2 and PPARγ activators to achieve the maximum protection against this oxidative stress will be very helpful in reducing the burden of many patients suffering from numerous oxidative stress-induced diseases, as an increasing amount of evidence strongly suggests the existence of a close association between the development of various metabolic disorders and drug-induced organ injuries, with oxidative stress^76,79^.

However, it is worth mentioning that the main limitation concerning the cardioprotective effect of TZDs is the generation of edema, which can predispose to congestive heart failure. Heart failure and edema, both of which are likely caused by renal salt retention, are significant side effects since they may limit the usage of TZDs^80^.

### Mixed activation of incretin and PPARγ, clinical significance and future perspectives

When the pharmacological effects of GLP-1 agonist were compared with TZDs, numerous shared properties were apparent including anti-inflammatory effects on endothelium. Using TNF-α-induced damage to human umbilical vein endothelial cells, an investigation of PPARγ transcriptional activity in the presence of exendin-4, a GLP-1RA, revealed that exendin-4 activation of PPARγ activity may partially explain the anti-inflammatory benefits of GLP-1. This suggests a clear connection between endothelial cell protection and a GLP-1 receptor agonist’s PPARγ-enhancing effect^81^.

Furthermore, GLP-1RAs have reported to induce PPARγ *via* activation of PKA, an important downstream substance of GLP-1 signaling^82^. On the other hand, evaluation of proglucagon regulation and GLP-1 release showed that activation of PPARβ/*δ* by synthetic agonists increase proglucagon expression and enhance bile acid and glucose-induced GLP-1 release by intestinal L cells. This confirms that therapeutic targeting of PPARβ/*δ* is considered as a hopeful strategy for the management of patients with T2DM, especially when combined with DPP-4is^83^.

It has been recently demonstrated that GLP-1RAs have anti-inflammatory effects on endothelial cells. For instance, GLP-1 receptor agonists prevented endothelial cell death, NF-kB activation, and NADPH oxidase upregulation brought on by TNFα. Atherosclerosis would be prevented as a result.

One of the primary causes of IR is the decreased phosphorylation level of IRS-1 serine, mainly as a result of PPARγ activity, which inhibits intracellular serine/threonine kinases like JNK^82^. Regarding Nrf2/Keap1 signaling pathway, its activation may occur through both PI3K/AKT incretin mediated and AMPK-PPAR-adiponectin (PPAR-mediated); therefore, synergistic activation of incretin and PPAR enhances Nrf2- dependent cytoprotecting potential by maintaining homeostasis and mitochondrial integrity^82^.

The convergence of incretin-based signaling and PPARγ activation represents a mechanistically coherent strategy for modulating metabolic homeostasis and redox balance. Increasing evidence from experimental and clinical studies suggests that dual engagement of these pathways produces effects that extend beyond glycemic control, particularly through coordinated regulation of the KEAP1-Nrf2 axis and downstream cytoprotective programs^84,85^.

Incretin hormones, primarily GLP-1, exert pleiotropic effects through G protein-coupled receptor activation, leading to cAMP accumulation and subsequent engagement of PI3K/AKT signaling. This cascade not only enhances insulin secretion but also intersects with redox-regulatory pathways^86,87^. A critical mechanistic node lies in AKT-mediated inhibition of glycogen synthase kinase-3β (GSK-3β), which suppresses the β-TrCP-dependent, KEAP1-independent degradation of Nrf2. As a result, incretin signaling facilitates Nrf2 stabilization even in the absence of canonical oxidative triggers. This mechanism provides a plausible explanation for the observed antioxidant and anti-inflammatory effects of GLP-1 receptor agonists reported in both preclinical models and clinical settings^88,89^.

In parallel, PPARγ activation, whether pharmacological or mediated indirectly through adiponectin signaling, engages the AMPK axis, thereby influencing mitochondrial function and cellular energy sensing. AMPK activation induces a metabolic shift characterized by enhanced fatty acid oxidation, improved mitochondrial biogenesis *via* PGC-1α, and suppression of mTORC1-driven anabolic processes. Importantly, this metabolic reprogramming alters intracellular redox status, increasing the likelihood of electrophilic modification of KEAP1 cysteine residues. Such modifications weaken KEAP1-mediated repression of Nrf2, promoting its nuclear translocation and transcriptional activation of AREs^90–92^.

The integration of these pathways is not merely additive but appears to be synergistic at multiple regulatory levels. Incretin-driven PI3K/AKT signaling and PPARγ-AMPK activation converge on both canonical (KEAP1-dependent) and non-canonical (GSK-3β/β-TrCP–mediated) mechanisms of Nrf2 regulation. This dual control enhances the strength of Nrf2 activation, ensuring sustained transcription of genes involved in glutathione synthesis, NADPH regeneration, and mitochondrial protection. Such coordinated regulation is particularly relevant in metabolic diseases characterized by chronic oxidative stress, including T2DM and obesity^89,93^.

Clinically, this convergence may explain the emerging benefits observed with combination of dual-acting therapies. For instance, a potential co-administration of incretin-based drugs with PPARγ agonists, or the development of single molecules with dual activity, may be associated with improved insulin sensitivity, reduced inflammatory markers, and attenuation of oxidative damage^94,95^. However, these benefits must be interpreted cautiously. Chronic or excessive activation of Nrf2 has been implicated in tumorigenesis and chemoresistance, particularly in contexts where KEAP1 function is compromised. Therefore, the therapeutic window for sustained Nrf2 activation remains a critical consideration^79,96^.

Another limitation lies in the differential tissue-specific responses to these pathways. While adipose tissue and liver show pronounced metabolic improvements, the effects in other organs, such as the brain or cardiovascular system, are less predictable and may depend on local signaling context, receptor expression, and disease state. Furthermore, PPARγ agonists are associated with adverse effects including weight gain, fluid retention, and potential cardiovascular risks, which complicates their integration into long-term therapeutic strategies^97,98^. Table 2 illustrates the shared pathways by which incretin-based agents and PPARγ agonists contribute to cardiovascular protection.

**Table 2:** Comparative mechanisms of incretin-based drugs and PPARγ agonists in modulating the Nrf-2-NFkB-adipokine axis.

**Mechanism/Effect**	**GLP-1 RAs/DPP-4is**	**PPARγ agonists**	**Shared pathways**
**Primary Action**	Enhance insulin secretion, inhibit glucagon	Improve insulin sensitivity, lipid uptake	Improved glucose homeostasis
**Nrf2 Activation**	Increase via cAMP/PI3K/PKC	Increase via transcriptional induction	Both enhance antioxidant response
**NF-κB Inhibition**	Increase cytokine-induced activation	Increase TNF-α–induced activation	Reduced inflammation
**Adipokine Modulation**	Increase Adiponectin, reduce resistin and leptin	Increase adiponectin, reduce resistin	Balanced adipokine profile
**Endothelial Protection**	Increase NO production, reduce oxidative injury	Increase NO bioavailability, reduce VSMC proliferation	Improved vascular function
**Clinical CV Outcome**	Reduce MACE (LEADER, SUSTAIN-6, REWIND)	Mixed (PROactive benefit, edema risk)	Cardioprotection potential
**Adverse Effects**	GI symptoms, rare pancreatitis	Weight gain, edema, HF risk	Require individualized therapy

Significant cardiovascular outcome trials, such as LEADER, SUSTAIN-6, and PROactive, demonstrate the clinical advantages of incretin-based therapies and PPARγ agonists in mitigating cardiovascular risk. Although these trials were not intended to assess Nrf2 signaling or adipokine modulation, the cardioprotective effects observed correspond with an increasing body of experimental and translational evidence indicating the convergence of incretin and PPARγ pathways on the Nrf2-adipokine axis. This convergence may enhance cardiovascular protection by reducing oxidative stress and inflammation; however, these mechanistic interpretations should be approached with caution and necessitate direct validation in specialized mechanistic and biomarker-focused studies^99–101^.

Future research should focus on refining this dual-target approach by identifying selective modulators that preserve beneficial metabolic and redox effects while minimizing adverse outcomes. One promising direction involves biased agonism at the GLP-1 receptor or selective PPARγ modulators (SPPARMs), which may decouple insulin-sensitizing effects from undesirable side effects. Additionally, deeper characterization of the AMPK-Nrf2 interface and its interaction with mitochondrial quality control pathways could reveal novel intervention points^102,103^.

At a systems level, the KEAP1-Nrf2 pathway emerges as a central integrative focus that links nutrient sensing, hormonal signaling, and oxidative stress responses. Mixed activation of incretin and PPARγ pathways forces this focus, offering a mechanistically grounded framework for therapeutic innovation. However, translating this concept into safe and effective clinical applications will require careful balancing of pathway activation, context-specific targeting, and long-term outcome evaluation^104,105^.

From a translational perspective, selective combinations of PPARγ and incretin agonists may amplify endogenous antioxidant defense while limiting adverse effects such as fluid retention^106^. Additionally, adiponectin, TNF-α, resistin and other mediators of the axis may serve as circulating biomarkers to monitor response to therapy and identify risks and benefits. However, long-term human studies are required to validate these options, especially focusing on the inter-individual variability and genetic polymorphism of adipokines profile.

## Conclusion

PPARγ and incretin-based therapies share overlapping antioxidant and anti-inflammatory actions *via* the Nrf2-NFkB-adipokine axis, offering cardiovascular protection, beyond glycemic control. Their ability to restore redox balance and to improve adipokine profiles highlight their potential in preserving vascular integrity and providing cardiac protection.

### Authors’ contribution

**Conceptualization:** Israa O. Kashmoola

**Literature screening, manuscript refinement, providing scientific oversight:** Shatha H. Mohammad and Mohammad H. Alsaaty

**Writing –Original draft preparation:** Israa O. Kashmoola

**Writing –Review and Editing:** Shatha H. Mohammad and Mohammad H. Alsaaty

## References

[1] Chen X, Xie N, Feng L (2025). Oxidative stress in diabetes mellitus and its complications: from pathophysiology to therapeutic strategies. Chin Med J (Engl).

[2] Caturano A, Rocco M, Tagliaferri G (2025). Oxidative stress and cardiovascular complications in type 2 diabetes: From pathophysiology to lifestyle modifications. Antioxidants.

[3] Hammad M, Raftari M, Cesário R (2023). Roles of oxidative stress and Nrf2 signaling in pathogenic and non-pathogenic cells: A possible general mechanism of resistance to therapy. Antioxidants.

[4] Feijóo-Bandín S, Aragón-Herrera A, Moraña Fernández S (2020). Adipokines and inflammation: Focus on cardiovascular diseases. Int J Mol Sci.

[5] Pan X, Xu S, Li J, Tong N (2020). The effects of DPP-4 inhibitors, GLP-1RAs, and SGLT-2/1 inhibitors on heart failure outcomes in diabetic patients with and without heart failure history: Insights from CVOTs and drug mechanism. Front Endocrinol (Lausanne).

[6] Yin L, Wang L, Shi Z, Ji X, Liu L (2022). The role of peroxisome proliferator-activated receptor gamma and atherosclerosis: post-translational modification and selective modulators. Front Physiol.

[7] Martín-Timón I (2014). Type 2 diabetes and cardiovascular disease: Have all risk factors the same strength?. World J Diabetes.

[8] Yilmaz S, Canpolat U, Aydogdu S, Abboud HE (2015). Diabetic cardiomyopathy; Summary of 41 years. Korean Circ J.

[9] De Geest B, Mishra M (2022). Role of oxidative stress in diabetic cardiomyopathy. Antioxidants.

[10] Kayama Y, Raaz U, Jagger A (2015). Diabetic cardiovascular disease induced by oxidative stress. Int J Mol Sci.

[11] Markova TN, Mishchenko NK, Petina D.V (2021). Adipocytokines: Modern definition, classification and physiological role. Probl Endocrinol.

[12] Tereshchenko I.V, Kamenskikh YA, Suslina AA (2016). Adiponectin in health and disease. Ter Arkh.

[13] Khoramipour K, Chamari K, Hekmatikar AA (2021). Adiponectin: Structure, physiological functions, role in diseases, and effects of nutrition. Nutrients.

[14] Fontanella RA, Scisciola L, Rizzo MR (2021). Adiponectin related vascular and cardiac benefits in obesity: Is there a role for an epigenetically regulated mechanism?. Front Cardiovasc Med.

[15] Shibata R, Ouchi N, Murohara T (2009). Adiponectin and cardiovascular disease. Circ J.

[16] Huber K, Szerenos E, Lewandowski D, Toczylowski K, Sulik A (2023). The role of adipokines in the pathologies of the central nervous system. Int J Mol Sci.

[17] Wysocka MB, Pietraszek-Gremplewicz K, Nowak D (2018). The role of apelin in cardiovascular diseases, obesity and cancer. Front Physiol.

[18] Castan-Laurell I, Dray C, Valet P (2021). The therapeutic potentials of apelin in obesity-associated diseases. Mol Cell Endocrinol.

[19] Antushevich H, Wójcik M Review: Apelin in disease. Clin Chim Acta.

[20] Daiber A, Xia N, Steven S (2019). New therapeutic implications of endothelial nitric oxide synthase (eNOS) function/dysfunction in cardiovascular disease. Int J Mol Sci.

[21] Hussein AA, Ahmed NA, Sakr HI, Atia T, Ahmed OM (2024). Omentin roles in physiology and pathophysiology: An up-to-date comprehensive review. Arch Physiol Biochem.

[22] Pan J, Baker KM (2007). Retinoic acid and the heart.

[23] Berry DC, Noy N (2009). All-trans-retinoic acid represses obesity and insulin resistance by activating both peroxisome proliferation-activated receptor β/*δ* and retinoic acid receptor. Mol Cell Biol.

[24] Kiernan K, MacIver NJ (2021). The role of the adipokine leptin in immune cell function in health and disease. Front Immunol.

[25] Poetsch MS, Strano A, Guan K (2020). Role of leptin in cardiovascular diseases. Front Endocrinol (Lausanne).

[26] Kirichenko TV, Markina YV, Bogatyreva AI, Tolstik TV, Varaeva YR, Starodubova AV (2022). The role of adipokines in inflammatory mechanisms of obesity. Int J Mol Sci.

[27] Jamaluddin MS, Weakley SM, Yao Q, Chen C (2012). Resistin: Functional roles and therapeutic considerations for cardiovascular disease. Br J Pharmacol.

[28] Abdalla MMI (2022). Role of visfatin in obesity-induced insulin resistance. World J Clin Cases.

[29] Kadoglou NPE, Gkontopoulos A, Kapelouzou A (2011). Serum levels of vaspin and visfatin in patients with coronary artery disease—Kozani study. Clin Chim Acta.

[30] Yue G, An Q, Xu X (2023). The role of Chemerin in human diseases. Cytokine.

[31] Yan Q, Zhang Y, Hong J (2012). The association of serum chemerin level with risk of coronary artery disease in Chinese adults. Endocrine.

[32] Liu J, Wang Y (2025). The potential role of retinol-binding protein 4 in heart failure: A review. Rev Cardiovasc Med.

[33] Barchetta I, Cimini FA, Dule S, Cavallo MG (2022). Dipeptidyl Peptidase 4 (DPP4) as a novel adipokine: Role in metabolism and fat homeostasis. Biomedicines.

[34] Chen SY, Kong XQ, Zhang KF, Luo S, Wang F, Zhang JJ (2022). DPP4 as a potential candidate in cardiovascular disease. J Inflamm Res.

[35] Sarkar J, Nargis T, Tantia O, Ghosh S, Chakrabarti P (2019). Increased Plasma Dipeptidyl Peptidase-4 (DPP4) activity is an obesity-independent parameter for glycemic deregulation in type 2 diabetes patients. Front Endocrinol (Lausanne).

[36] Silva LB, dos Santos Neto AP, Maia SMAS (2019). The role of TNF-α as a proinflammatory cytokine in pathological processes. Open Dent J.

[37] Ridker PM, Rane M (2021). Interleukin-6 signaling and Anti-Interleukin-6 therapeutics in cardiovascular disease. Circ Res.

[38] Akbari M, Hassan-Zadeh V (2018). IL-6 signalling pathways and the development of type 2 diabetes. Inflammopharmacology.

[39] Marques FZ, Prestes PR, Byars SG (2017). Experimental and human evidence for Lipocalin-2 (Neutrophil Gelatinase-Associated Lipocalin [NGAL]) in the development of cardiac hypertrophy and heart failure. J Am Heart Assoc.

[40] Elkhidir AE, Eltaher HB, Mohamed AO (2017). Association of lipocalin-2 level, glycemic status and obesity in type 2 diabetes mellitus. BMC Res Notes.

[41] Clemente-Suárez VJ, Redondo-Flórez L, Beltrán-Velasco AI (2023). The role of adipokines in health and disease. Biomedicines.

[42] Hemat Jouy S, Mohan S, Scichilone G, Mostafa A, Mahmoud AM (2024). Adipokines in the crosstalk between adipose tissues and other organs: Implications in cardiometabolic diseases. Biomedicines.

[43] Smekal A, Vaclavik J (2017). Adipokines and cardiovascular disease: A comprehensive review. Biomed Pap.

[44] Satta S, Mahmoud AM, Wilkinson FL, Yvonne Alexander M, White SJ (2017). The role of Nrf2 in cardiovascular function and disease. Hrelia S, ed. Oxid Med Cell Longev.

[45] Wu X, Wei J, Yi Y, Gong Q, Gao J (2022). Activation of Nrf2 signaling: A key molecular mechanism of protection against cardiovascular diseases by natural products. Front Pharmacol.

[46] He F, Ru X, Wen T (2020). NRF2, a transcription factor for stress response and beyond. Int J Mol Sci.

[47] Zgorzynska E, Dziedzic B, Walczewska A (2021). An overview of the Nrf2/ARE pathway and its role in neurodegenerative diseases. Int J Mol Sci.

[48] Zang H, Mathew RO, Cui T (2020). The dark side of Nrf2 in the heart. Front Physiol.

[49] Wu J, Sun X, Jiang Z (2020). Protective role of NRF2 in macrovascular complications of diabetes. J Cell Mol Med.

[50] Yang JJ, Tao H, Hu W (2014). MicroRNA-200a controls Nrf2 activation by target Keap1 in hepatic stellate cell proliferation and fibrosis. Cell Signal.

[51] Howden R (2013). Nrf2 and cardiovascular defense. Oxid Med Cell Longev.

[52] Steven S, Frenis K, Oelze M (2019). Vascular inflammation and oxidative stress: Major triggers for cardiovascular disease. Oxid Med Cell Longev.

[53] Li W, Khor TO, Xu C (2008). Activation of Nrf2-antioxidant signaling attenuates NFκB-inflammatory response and elicits apoptosis. Biochem Pharmacol.

[54] Smith R, Tran K, Smith C, McDonald M, Shejwalkar P, Hara K (2016). The role of the Nrf2/ARE antioxidant system in preventing cardiovascular diseases. Diseases.

[55] García-García VA, Alameda JP, Page A, Casanova ML (2021). Role of NF-κB in ageing and age-related diseases: Lessons from genetically modified mouse models. Cells.

[56] Zhou S, Sun W, Zhang Z, Zheng Y (2014). The role of Nrf2-mediated pathway in cardiac remodeling and heart failure. Oxid Med Cell Longev.

[57] Chen QM (2021). Nrf2 for cardiac protection: pharmacological options against oxidative stress. Trends Pharmacol Sci.

[58] Gutiérrez-Cuevas J, Galicia-Moreno M, Monroy-Ramírez HC (2022). The role of NRF2 in obesity-associated cardiovascular risk factors. Antioxidants.

[59] Rodríguez C, Muñoz M, Contreras C, Prieto D (2021). AMPK, metabolism, and vascular function. FEBS J.

[60] Ren Y, Zhao H, Yin C (2022). Adipokines, hepatokines and myokines: focus on their role and molecular mechanisms in adipose tissue inflammation. Front Endocrinol (Lausanne).

[61] Baggio LL, Drucker DJ (2007). Biology of incretins: GLP-1 and GIP. Gastroenterology.

[62] Zheng Z, Zong Y, Ma Y (2024). Glucagon-like peptide-1 receptor: mechanisms and advances in therapy. Signal Transduct Target Ther.

[63] Yaribeygi H, Maleki M, Butler AE, Jamialahmadi T, Sahebkar A (2021). The impact of incretin-based medications on lipid metabolism, Sasaoka T, ed. J Diabetes Res.

[64] Burgmaier M, Heinrich C, Marx N (2013). Cardiovascular effects of GLP-1 and GLP-1-based therapies: implications for the cardiovascular continuum in diabetes?. Diabet Med.

[65] Solini A, Tricò D, Prato S.Del (2023). Incretins and cardiovascular disease: to the heart of type 2 diabetes?. Diabetologia.

[66] Król M, Kupnicka P, Zychowska J (2025). Molecular insights into the potential cardiometabolic effects of GLP-1 receptor analogs and DPP-4 inhibitors. Int J Mol Sci.

[67] Aristizábal-Colorado D, Corredor-Rengifo D, Sierra-Castillo S (2025). A decade of progress in type 2 diabetes and cardiovascular disease: advances in SGLT2 inhibitors and GLP-1 receptor agonists –a comprehensive review. Front Endocrinol (Lausanne).

[68] Marx N, Husain M, Lehrke M, Verma S, Sattar N (2022). GLP-1 receptor agonists for the reduction of atherosclerotic cardiovascular risk in patients with Type 2 diabetes. Circulation.

[69] Iorga R, Bacalbasa N, Carsote M (2020). Metabolic and cardiovascular benefits of GLP-1 agonists, besides the hypoglycemic effect (Review). Exp Ther Med.

[70] Nuamnaichati N, Mangmool S, Chattipakorn N, Parichatikanond W (2020). Stimulation of GLP-1 receptor inhibits Methylglyoxal-Induced Mitochondrial dysfunctions in H9c2 cardiomyoblasts: potential role of Epac/PI3K/Akt pathway. Front Pharmacol.

[71] Zhou H, Yang J, Xin T (2014). Exendin-4 protects adipose-derived mesenchymal stem cells from apoptosis induced by hydrogen peroxide through the PI3K/Akt–Sfrp2 pathways. Free Radic Biol Med.

[72] Changizi Z, Kajbaf F, Moslehi A (2023). an overview of the role of peroxisome proliferator-activated receptors in liver diseases. J Clin Transl Hepatol.

[73] Abbas A, Blandon J, Rude J, Elfar A, Mukherjee D (2012). PPAR- & gamma; agonist in treatment of diabetes: cardiovascular safety considerations. Cardiovasc Hematol Agents Med Chem.

[74] Zhu M, Flynt L, Ghosh S (2011). Anti-inflammatory effects of thiazolidinediones in human airway smooth muscle cells. Am J Respir Cell Mol Biol.

[75] Giby VG, Ajith TA (2014). Role of adipokines and peroxisome proliferator-activated receptors in nonalcoholic fatty liver disease. World J Hepatol.

[76] Giby VG, Ajith TA (2014). Role of adipokines and peroxisome proliferator-activated receptors in nonalcoholic fatty liver disease. World J Hepatol.

[77] Polvani S, Tarocchi M, Galli A (2012). PPAR and oxidative stress: Con(β) catenating NRF2 and FOXO. PPAR Res.

[78] Huang J, Tabbi-Anneni I, Gunda V, Wang L (2010). Transcription factor Nrf2 regulates SHP and lipogenic gene expression in hepatic lipid metabolism. Am J Physiol Gastrointest Liver Physiol.

[79] Lee C (2017). Collaborative power of Nrf2 and PPAR γ activators against metabolic and drug-induced oxidative injury. Alexander MY, ed. Oxid Med Cell Longev.

[80] Horita S, Nakamura M, Satoh N, Suzuki M, Seki G (2015). Thiazolidinediones and edema: recent advances in the pathogenesis of thiazolidinediones-induced renal sodium retention. PPAR Res.

[81] Shiraki A, ichi Oyama.J, Komoda H (2012). The glucagon-like peptide 1 analog liraglutide reduces TNF-α-induced oxidative stress and inflammation in endothelial cells. Atherosclerosis.

[82] Onuma H, Inukai K, Kitahara A (2014). The glucagon-like peptide 1 receptor agonist enhances intrinsic peroxisome proliferator-activated receptor γ activity in endothelial cells. Biochem Biophys Res Commun.

[83] Daoudi M, Hennuyer N, Borland MG (2011). PPARβ/*δ* activation induces Enteroendocrine L cell GLP-1 production. Gastroenterology.

[84] Oh Y, Jun HS (2017). Effects of glucagon-like Peptide-1 on oxidative stress and Nrf2 signaling. Int J Mol Sci.

[85] Quarta C, Stemmer K, Novikoff A (2022). GLP-1-mediated delivery of tesaglitazar improves obesity and glucose metabolism in male mice. Nat Metab.

[86] Oh YS, Jun HS (2017). Effects of Glucagon-Like Peptide-1 on oxidative stress and Nrf2 signaling. Int J Mol Sci.

[87] Fernández-Millán E, Martín MA, Goya L (2016). Glucagon-like peptide-1 improves beta-cell antioxidant capacity via extracellular regulated kinases pathway and Nrf2 translocation. Free Radic Biol Med.

[88] Abdel Razek NS, Nassar NN, Sayed RH, El-Sahar AE, Abdallah DM (2025). Liraglutide orchestrates ferroptosis defense against murine cisplatin acute kidney injury: NRF2 activation via both KEAP1-dependent and -independent mechanisms is essential for SLC7A11/GPX4 renoprotection. J Trace Elem Med Biol.

[89] Salazar M, Rojo AI, Velasco D, de Sagarra RM, Cuadrado A (2006). Glycogen synthase Kinase-3β inhibits the xenobiotic and antioxidant cell response by direct phosphorylation and nuclear exclusion of the transcription factor Nrf2. J Biol Chem.

[90] Niu P, Zhang R, Zhang C, Li S, Li Y (2024). Identifying novel proteins for migraine by integrating proteomes from blood and <scp>CSF</scp>with genome-wide association data. CNS Neurosci Ther.

[91] Jantrapirom S, Piccolo L.Lo, Yamaguchi M (2019). Non-proteasomal UbL-UbA family of proteins in neurodegeneration. Int J Mol Sci.

[92] Hu Q, Yammani RD, Brown-Harding H, Soto-Pantoja DR, Poole LB, Lukesh JC (2022). Mitigation of doxorubicin-induced cardiotoxicity with an H2O2-Activated, H2S-Donating hybrid prodrug. Redox Biol.

[93] Hayes JD, Chowdhry S, Dinkova-Kostova AT, Sutherland C (2015). Dual regulation of transcription factor Nrf2 by Keap1 and by the combined actions of β-TrCP and GSK-3. Biochem Soc Trans.

[94] Wang S, Dougherty EJ, Danner RL (2016). PPARγ signaling and emerging opportunities for improved therapeutics. Pharmacol Res.

[95] Alnaser RI, Alassaf FA, Abed MN (2024). Incretin-based therapies: a promising approach for modulating oxidative stress and insulin resistance in sarcopenia. J Bone Metab.

[96] Jeddi F, Soozangar N, Sadeghi MR, Somi MH, Samadi N (2017). Contradictory roles of Nrf2/Keap1 signaling pathway in cancer prevention/promotion and chemoresistance. DNA Repair (Amst).

[97] Ciudin A, Hernandez C, Simo R (2012). Update on cardiovascular safety of PPARgamma agonists and relevance to medicinal chemistry and clinical pharmacology. Curr Top Med Chem.

[98] Kounatidis D, Vallianou NG, Rebelos E (2025). The many facets of PPAR-γ agonism in obesity and associated comorbidities: benefits, risks, challenges, and future directions. Curr Obes Rep.

[99] Dormandy JA, Charbonnel B, Eckland DJA (2005). Secondary prevention of macrovascular events in patients with type 2 diabetes in the PROactive Study (PROspective pioglitAzone Clinical Trial In macroVascular Events): a randomised controlled trial. Lancet (London, England).

[100] Marso SP, Bain SC, Consoli A (2016). Semaglutide and cardiovascular outcomes in patients with Type 2 Diabetes. N Engl J Med.

[101] Marso SP, Daniels GH, Brown-Frandsen K (2016). Liraglutide and cardiovascular outcomes in Type 2 Diabetes. N Engl J Med.

[102] Jangra A, Babu B, Divakar S (2025). An in-depth review of PPARγ modulators as anti-diabetes therapeutics. Drug Metab Rev.

[103] Lu X, Yang L (2026). Glucagon-like peptide-1 and dual/triple receptor agonists in the treatment of metabolic dysfunction-associated steatotic liver disease: advances in mechanistic research. Front Med.

[104] Li B, Ming H, Qin S (2025). Redox regulation: mechanisms, biology and therapeutic targets in diseases. Signal Transduct Target Ther.

[105] Hayes JD, Dinkova-Kostova AT (2014). The Nrf2 regulatory network provides an interface between redox and intermediary metabolism. Trends Biochem Sci.

[106] Hirukawa H, Kaneto H, Shimoda M (2015). Combination of DPP-4 inhibitor and PPARγ agonist exerts protective effects on pancreatic β-cells in diabetic db/db mice through the augmentation of IRS-2 expression. Mol Cell Endocrinol.

